# Antifungal potential of copper oxide nanoparticles against *Microsporum canis* isolates in canine and feline dermatophytosis

**DOI:** 10.22034/cmm.2025.345248.1604

**Published:** 2025-02-01

**Authors:** Javad Malakootikhah, Donya Nikaein, Hanieh Golchini, Alireza Khosravi

**Affiliations:** 1 Department of Nanobiotechnology, College of Interdisciplinary Science and Technologies, University of Tehran, Tehran, Iran; 2 Department of Microbiology and Immunology, Faculty of Veterinary Medicine, University of Tehran, Tehran, Iran

**Keywords:** Antifungal, Copper oxide nanoparticles, Dermatophytosis, Green synthesis, *Microsporum canis*

## Abstract

**Background and Purpose:**

Dermatophytosis, or ringworm, is a highly contagious fungal infection caused by dermatophytes, like *Microsporum canis*, which primarily affects cats and dogs and poses a significant zoonotic threat. Increasing prevalence of drug-resistant strains complicates the treatment of *M. canis* infections, necessitating the exploration of new therapeutic approaches. Nanotechnology, particularly copper oxide nanoparticles (CuO NPs), has emerged as a promising solution due to its potent antimicrobial properties and potential to overcome resistance. This study aimed to evaluate the antifungal efficacy of CuO NPs against *M. canis* isolates collected from companion animals. The goal was to develop more effective treatment options for dermatophytosis, addressing the need for alternative therapies and the challenge of antifungal resistance.

**Materials and Methods:**

CuO NPs were synthesized using a green chemistry approach, employing *Eichhornia crassipes* leaf extract. Concurrently, *M. canis* isolates were obtained from infected animals and cultured for purity. Antifungal activity of the CuO NPs against the isolates was assessed through disk diffusion and microdilution tests, and the results were statistically analyzed to confirm their significance. **Cell dens (*10**^5^*****

**Results:**

The synthesized CuO NPs exhibited high purity, small size, and cubic morphology. Statistical analysis of the disk diffusion and microdilution tests confirmed the significant antifungal efficacy of CuO NPs against *M. canis* isolates (ANOVA, *p*<0.05). Minimum inhibitory concentration (MIC) values ranged from 500 to 1,000 ppm, while minimum fungicidal concentration (MFC) values were between 1,000 and 2,000 ppm. Average MFC/MIC ratio of 2.6, confirmed through paired t-test (*p*=0.003), demonstrated the fungicidal properties of the CuO NPs.

**Conclusion:**

This study highlighted the potent antifungal capabilities of CuO NPs against *M. canis*, marking them as a promising alternative to conventional treatments. With further optimization and research, CuO NPs could revolutionize the management of dermatophytosis, offering a new frontier in combating drug-resistant fungal infections.

## Introduction

Dermatophytosis, commonly known as ringworm, is a contagious fungal infection that affects both humans and animals, particularly domestic pets, such as dogs and cats. The disease is caused by dermatophytes, a group of fungi that thrive on keratin, which is found in the skin, nails, and hair of both humans and animals [ 1 , 2 ]. Among the dermatophytes, *Microsporum canis* is the primary causative agent of dermatophytosis in cats and dogs. This zoophilic fungus is not only a significant pathogen in animals but also poses a zoonotic risk, with an increasing number of human infections reported worldwide. While *M. canis* is most frequently isolated from infected or carrier cats, it can also infect other mammals, including dogs, rabbits, rodents, and even humans, making it a severe public health concern [ 3 , 4 ].

Clinical presentation of *M. canis* infection varies between species. In cats, the symptoms range from multifocal alopecia, scaling, and miliary dermatitis to being completely asymptomatic. In dogs, the infection manifests as pustules, papules, and ring-form alopecia [ 5 ]. Despite the variety of clinical signs, the underlying pathophysiology of *M. canis* infection remains poorly understood. However, dermatophyte-secreted proteases are believed to play a crucial role in the adhesion and invasion of the stratum corneum and other keratinized structures of the epidermis, facilitating the establishment of infection [ 6 , 7 ].

Management of dermatophytosis is increasingly challenging due to emerging infections and the increasing drug resistance in pathogens, which compromise the efficacy of current antifungal treatments, such as Griseofulvin, Terbinafine, Itraconazole, and Fluconazole [ 1 , 8 , 9 ]. These limitations highlight the urgent need for innovative therapeutic approaches. In recent years, nanotechnology has emerged as a revolutionary field with the potential to address various challenges in applied sciences, including medicine [ 10 , 11 ]. Nanotechnology, particularly the use of copper oxide nanoparticles (CuO NPs), offers a promising solution. Due to its unique properties, including a high surface area-to-volume ratio, cost-effectiveness, and broad-spectrum antimicrobial activity, CuO NPs have become a focus in medicine [ 12 - 14 ]. Additionally, their chemical stability, compatibility with polymers, and tunable surface features enhance their potential as effective antimicrobial agents [ 15 - 17 ].

CuO NPs have emerged as a promising option for the treatment of animal fungal infections, including dermatophytosis caused by *M. canis*. Unique properties of CuO NPs, such as their small size and high surface area, contribute to their enhanced antimicrobial activity [ 14 ]. These NPs can disrupt fungal cell membranes, generate reactive oxygen species (ROS), and interfere with cellular processes essential for fungal survival. Due to these properties, CuO NPs have demonstrated significant efficacy against various fungi, including dermatophytes that cause ringworm in dogs and cats. Moreover, CuO NPs offer advantages over traditional antifungal treatments, such as better tissue penetration and a reduced likelihood of resistance development [ 18 - 20 ]. As a result, they hold great potential as a novel therapeutic strategy in veterinary medicine, offering a more effective and targeted approach to managing dermatophytosis in companion animals.

Given the rising concern over drug-resistant fungal infections and the promising antimicrobial properties of CuO NPs, this study aimed to evaluate the antifungal potential of CuO NPs against *M. canis* isolates collected from dogs and cats with dermatophytosis. Findings of this study could lead to the development of more effective treatments for dermatophytosis in veterinary medicine, addressing both the need for alternative therapies and the growing issue of antifungal resistance.

## Materials and Methods

*Eichhornia crassipes* plants, and all chemicals utilized in this experiment were obtained from Sigma-Aldrich Chemicals, India. The used culture mediums were sourced from Merck, Germany. The sterile blank paper discs were sourced from Padtan-Teb Company, Iran. The commercial itraconazole discs were sourced from Neosensitabs®, Denmark.

The laboratory glassware was soaked overnight in an acid-cleaning solution, followed by thorough rinsing with tap and distilled water. Milli-Q water was employed to synthesize NPs.

### Isolation and Preparation of M. canis Isolates

In total, 10 *M. canis* isolates were collected from dogs and cats diagnosed with dermatophytosis at the small animal hospital of the Faculty of Veterinary Medicine, University of Tehran, Tehran, Iran. Clinical signs of dermatophytosis were carefully recorded in dogs and cats. Skin scrapings and hair samples were obtained from the affected animals and inoculated on Sabouraud Dextrose Agar (SDA) supplemented with chloramphenicol and cycloheximide to inhibit bacterial and non-dermatophytic fungal growth. The plates were incubated at 28 °C for 2-4 weeks, during which time the growth of typical *M. canis* colonies was monitored.

Upon confirmation of *M. canis* identification by morphological examination and lactophenol cotton blue staining, the isolates were subcultured to obtain pure colonies. Afterward, these pure isolates were used for subsequent antifungal susceptibility testing [ 21 , 22 ]. In a prior study, these isolates were reverified using both biochemical and molecular methods [ 23 ].

### Synthesis and characterization of CuO NPs

The CuO NPs were synthesized using a green chemistry approach, which is environmentally friendly and minimizes the use of hazardous chemicals [ 14 , 24 , 25 ]. The synthesis process involved using *E. crassipes* leaf extract as a reducing and stabilizing agent. Fresh leaves were washed thoroughly with distilled water, air-dried, and finely chopped. A 10% (w/v) aqueous extract was prepared by boiling the chopped leaves in Milli-Q water for 30 min, followed by filtration through Whatman No. 1 filter paper to obtain a clear extract.

Copper sulfate pentahydrate (CuSO_4_.5H_2_O) was used as the precursor for the CuO NPs. A 0.1 M solution of copper sulfate was prepared and mixed with the leaf extract in a 1:1 ratio under constant stirring at 60 °C. The reaction mixture was kept at this temperature for 2 h until a color change from light blue to dark brown indicated the formation of CuO NPs. The NPs were then collected by centrifugation at 10,000 rpm for 15 min, washed several times with distilled water and ethanol to remove any impurities, and dried at 60 °C overnight. The final stock solution of CuO NPs was prepared at a concentration of 8,000 ppm for subsequent experiments.

The synthesized CuO NPs were characterized using several analytical techniques. The X-ray diffraction (XRD) was utilized to assess the elemental composition and purity of the NPs. Scanning electron microscopy (SEM) was employed to determine the size distribution and morphology of the CuO NPs. The XRD analysis was conducted to investigate the crystalline structure of the NPs.

### Disk diffusion test

The pure *M. canis* isolates were prepared for disk diffusion assay to evaluate the antifungal activity of CuO NPs. For the disk diffusion test, a conidial suspension of each isolate was adjusted to a concentration of 2.5 × 10^3^ conidia/mL using a hemocytometer [ 23 , 26 ]. Sterile cotton swabs were used to inoculate the suspensions evenly onto SDA plates. Sterile blank paper disks impregnated with varying concentrations of CuO NPs (from 4,000 ppm to 250 ppm) were then placed on the inoculated plates, incubating at 28 °C for seven days. Additionally, commercial discs containing 10 μg of itraconazole were used as a control to compare the antifungal effectiveness of the CuO NPs against *M. canis* isolates. The inhibition zones around each disk were measured to assess antifungal activity. All experiments were conducted in triplicate.

### Microdilution Test

The microdilution test was conducted to determine the minimum inhibitory concentration (MIC) and minimum fungicidal concentration (MFC) of CuO NPs against the *M. canis* isolates, following the microdilution method as outlined in the CLSI M38-A2 guidelines [ 27 ]. Serial two-fold dilutions of the stock solution were prepared in RPMI 1640 medium to achieve concentrations ranging from 8000 ppm to 15.625 ppm.

Each well of a 96-well microtiter plate was inoculated with 100 µL of the *M. canis* conidial suspension (2.5 × 10^3^ conidia/mL) and 100 µL of the CuO NP dilutions. The plates were incubated at 28°C for 72 hours. The MIC was determined to have the lowest concentration of CuO NPs, which completely inhibited visible fungal growth, as assessed by visual inspection.

To determine the MFC, 100 µL from wells showing no visible growth (including and above the MIC) were subcultured onto SDA plates. The plates were incubated at 28°C for an additional seven days. The MFC was defined as the lowest concentration of CuO NPs at which no fungal growth was observed on the agar plates, indicating a fungicidal effect. All experiments were conducted in triplicate.

The MFC/MIC ratio was calculated to evaluate the fungicidal versus fungistatic nature of CuO NPs against the M. canis isolates. The ratio is determined by dividing the MFC by the MIC. A low MFC/MIC ratio (≤4) indicates that the agent is fungicidal, meaning it kills the fungi, whereas a higher ratio (>4) suggests a fungistatic effect, where the agent inhibits fungal growth but does not kill the organisms [ 28 ]. This ratio is crucial in understanding the efficacy of CuO NPs as a potential antifungal treatment, as it helps differentiate between concentrations that merely suppress growth and those that effectively eliminate the fungal pathogens.

### Statistical Analysis

Descriptive statistics, including mean and standard deviation, were calculated for all quantitative variables. The differences in antifungal efficacy between the CuO NPs and the control (Itraconazole) were assessed using analysis of variance (ANOVA) followed by post hoc tests for multiple comparisons. The MIC and MFC values were compared using the paired t-test. A p-value of less than 0.05 was considered statistically significant. All statistical analyses were performed using SPSS software version 26.0.

## Results

### Morphological Characteristics of M. canis Isolates

In total, 10 isolates of *M. canis* were obtained from dogs and cats diagnosed with dermatophytosis. The colonies displayed typical *M. canis* characteristics, including a white to cream-colored, fluffy, and cottony texture with a yellow to orange pigmentation on the reverse side. Microscopically, the isolates exhibited numerous spindle-shaped, rough-walled macroconidia with thick septa and pointed ends, which are the diagnostic features of *M. canis*. Additionally, a few microconidia, pyriform in shape, were observed scattered among the hyphae.

In addition to the characteristic colony morphology and microscopic examination, the isolates demonstrated strong positive reactions in the hair perforation test, a distinctive feature of *M. canis*. The isolates also exhibited fluorescence under Wood's lamp due to pteridine, a compound commonly associated with *M. canis* infections. These diagnostic features collectively confirmed the accurate identification and isolation of *M. canis* from the clinical samples.

### Clinical signs in dogs and cats with dermatophytosis

The dogs and cats from which the *M. canis* isolates were obtained exhibited various clinical signs consistent with dermatophytosis. Infected cats presented with multifocal alopecia, scaling, and crusting, particularly around the face, ears, and limbs. Some cats were asymptomatic carriers, showing no visible signs despite being culture-positive. In dogs, the clinical manifestations included circular areas of alopecia with erythema, scaling, and occasional pustules and papules. Lesions were most commonly observed on the face, paws, and tail. Severity of the signs varied, with some animals showing mild localized lesions while others had more extensive involvement.

### Characterization of CuO NPs

Size of the NPs ranged from 10 to 30 nm, as observed through SEM imaging, which also revealed their welldefined cubic morphology .

**Figure 1:**
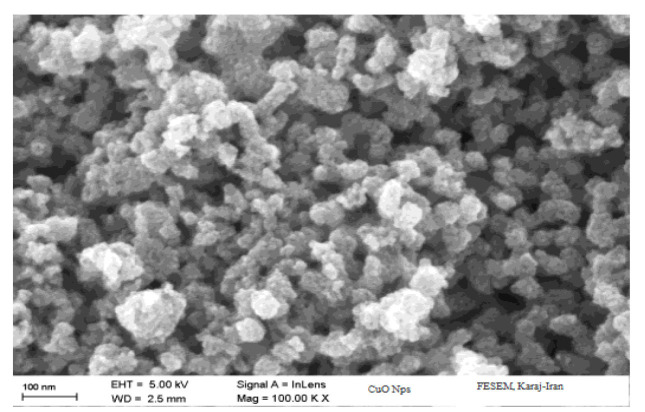
Scanning electron microscopy image showing the morphology and size distribution of the synthesized copper oxide nanoparticles. The image reveals the cubic shape, size, and uniformity of the nanoparticles.

Size of the NPs ranged from 10 to 30 nm, as observed through SEM imaging, which also revealed their well-defined cubic morphology (Figure 1). The NPs were green in color and maintained a stable liquid form, suitable for further applications.

**Figure 2:**
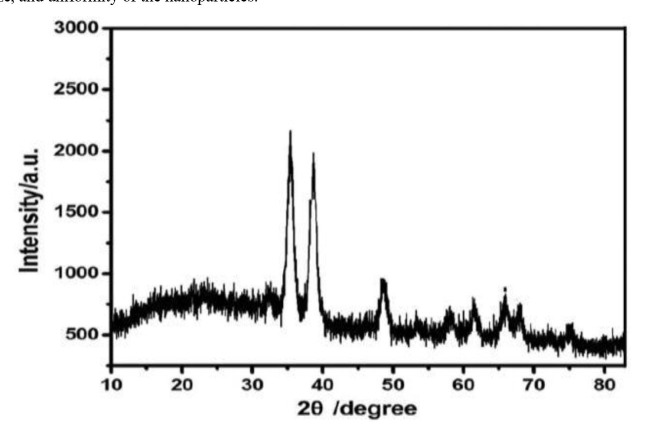
The X-ray diffraction (XRD) pattern for analyzing the crystal structure of synthesized copper oxide nanoparticles (CuO NPs). The captured XRD diagram displays the characteristic diffraction peaks used to determine the phase purity and crystalline structure of the CuO NPs.

The XRD analysis was performed to determine the crystalline structure of the CuO NPs (Figure 2). The XRD pattern displayed sharp and distinct peaks, consistent with the cubic phase of CuO, and confirmed the high crystallinity of the synthesized NPs. The diffraction peaks corresponded well with the standard reference data for CuO, further validating the successful synthesis of pure and crystalline NPs. These characteristics suggest that the CuO NPs produced by the green synthesis method are of high quality and suitable for antifungal applications, as their small size, cubic morphology, and high purity are crucial for enhancing their interaction with microbial cells.

### Disk diffusion test results

**Figure 3:**
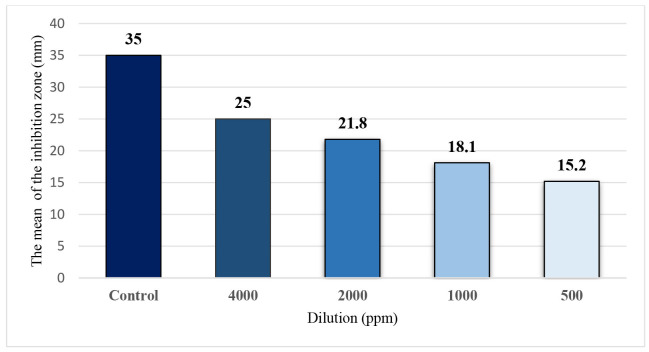
Mean diameter of growth inhibition zones at various dilutions of copper oxide nanoparticles, compared to the positive control (itraconazole, 10 μg).

The disk diffusion test results showed that the highest level of fungal growth inhibition by CuO NPs occurred at a concentration of 4,000 ppm (Figure 3). The NPs effectively inhibited fungal growth down to a concentration of 500 ppm, though the degree of inhibition decreased with lower concentrations. At 250 ppm, no significant growth inhibition was observed. However, across all concentrations, the antifungal activity of CuO NPs was less pronounced, compared to the control group treated with itraconazole. Statistically, there was no significant difference among the 10 *M. canis* isolates tested in terms of the antifungal effects of CuO NPs.

### Microdilution test results

The statistical analysis and microdilution results demonstrated that CuO NPs exhibited significant antifungal efficacy against *M. canis* isolates. The MIC values (500-1,000 ppm) and MFC values (1,000-2,000 ppm) indicated that CuO NPs were effective at both inhibition of fungal growth and achievement of complete fungal cell death (Table 1). Statistical comparisons, including ANOVA (*p*<0.05) and paired t-tests (*p*=0.003), revealed that CuO NPs significantly outperformed the control (itraconazole), highlighting their potential as a more effective antifungal agent.

**Table 1:** Microdilution test results.

Isolates	MIC of CuO NPs (ppm)	MFC of CuO NPs (ppm)	MIC of itraconazole (µg/ml)	MFC of itraconazole (µg/ml)	MFC/MIC ratio
1	500	1000	0.75	1.5	2
2	666	1000	0.75	1.5	2
3	500	2000	0.75	1.5	4
4	500	1000	0.75	1.5	2
5	1000	2000	0.75	1.5	2
6	500	1000	0.75	1.5	2
7	500	1000	0.75	1.5	2
8	666	2000	0.75	1.5	4
9	500	1000	0.75	1.5	2
10	666	2000	0.75	1.5	4
Mean	600	1117	0.75	1.5	2.6

The values represent the mean of three replicates for each test. MIC: minimum inhibitory concentration, MFC: minimum fungicidal concentration.

### MFC/MIC ratio

The MFC/MIC ratio was calculated to evaluate the fungicidal properties of the CuO NPs. The MFC/MIC values were equal to or less than four, with an average ratio of 2.6 (Table 1). This ratio confirmed that the CuO NPs exhibited fungicidal activity, killing the fungal cells rather than merely inhibiting their growth.

## Discussion

The discussion begins with the most prominent findings of the study, highlighting the significant antifungal potential of CuO NPs against M. canis isolates obtained from dogs and cats with dermatophytosis. Social life, contact with animals, usage of antibiotics, corticosteroids, and anti-cancer medications, as well as other factors contribute to fungal diseases, including dermatophytosis. Although various chemical drugs with different formulations have been employed to treat dermatophytosis, their chemical natures, side effects, and high costs necessitate alternative methods to control fungal diseases. An increase in the prevalence of infectious diseases and the rapid spread of resistance among pathogenic bacteria and fungi against current agents are significant concerns. Despite increased awareness and the development of new treatments, the prevalence and mortality rates associated with microbial infections remain high [ 29 - 31 ].

Results of the current study provide valuable insights into the antifungal efficacy of CuO NPs, especially given the consistency of the results across all three tests, namely MIC, MFC, and Disk Diffusion.

Advancement of nanotechnology has opened new avenues for combating antifungal resistance and improving the treatment of fungal infections, even with lower doses of antimicrobial agents [ 32 ]. Metal NPs, such as calcium, magnesium, zinc, and CuO have demonstrated significant antimicrobial properties. Specifically, CuO NPs have garnered attention due to their unique characteristics, such as high surface area-to-volume ratio and the ability to disrupt microbial cell walls [ 33 , 34 ]. Findings of the present study suggest that CuO NPs could serve as an effective alternative to conventional antifungal agents, potentially reducing the required dosage and minimizing associated side effects while addressing the growing issue of antifungal resistance.

Characterization of the synthesized CuO NPs revealed their high purity, small size, and cubic morphology, which are critical factors contributing to their antifungal activity. The XRD analysis confirmed the crystalline structure of the NPs, which is consistent with the cubic phase of CuO. Results of this study are consistent with earlier research conducted by Alizadeh and Ebrahimzadeh, Koteeswari et al., Shanan et al., and Phang et al. [ 35 - 38 ].

The present study synthesized CuO NPs using a green synthesis method, offering several advantages over traditional chemical synthesis. Green synthesis employs natural reducing agents from plants and microorganisms, making it a more eco-friendly and sustainable approach. This method is not only simpler and faster but also cost-effective and environmentally friendly, avoiding the use of hazardous chemicals. Additionally, green synthesis eliminates the need for complex and time-consuming processes associated with the maintenance of cell cultures, which is especially beneficial for large-scale production of NPs. Plant extracts are particularly advantageous in this context, as they are easier to handle and offer a broad range of bioactive compounds that enhance the antimicrobial, anti-cancer, antidiabetic, anti-inflammatory, and antioxidant properties of the resulting NPs [ 39 , 40 ]. As demonstrated in various studies, including a study performed by Thiruvengadam et al., NPs synthesized from plants exhibit excellent biological activities, making them highly suitable for medical applications [ 41 ]. Usage of plant-based green synthesis aligns with the growing need for environmentally friendly production methods and enhances the therapeutic potential of NPs, offering a promising alternative for combating diseases [ 40 ].

Antifungal mechanism of CuO NPs is multifaceted, involving several pathways that collectively disrupt fungal cell function and viability. One of the primary mechanisms is the generation of ROS upon interaction with fungal cells. These ROS, including hydroxyl radicals and superoxide anions, cause oxidative stress, damaging cellular components, such as lipids, proteins, and DNA. Another mechanism involves the disruption of fungal cell membrane integrity. The CuO NPs can interact directly with the cell membrane, leading to increased permeability and subsequent leakage of intracellular contents. This membrane disruption is facilitated by the small size and high surface reactivity of the NPs, which allow them to penetrate and destabilize the lipid bilayer effectively [ 42 - 45 ].

Additionally, CuO NPs can interfere with the enzymatic activities within fungal cells by binding to thiol groups in proteins, thereby inhibiting essential metabolic processes. This disruption in enzymatic function can lead to cellular apoptosis and death. Studies have also shown that CuO NPs can enter fungal cells and accumulate within, leading to further internal damage. These mechanisms collectively contribute to the potent antifungal activity observed in CuO NPs, making them a promising alternative for the treatment of fungal infections, particularly in cases involving resistance to conventional antifungal drugs [ 43 , 46 , 47 ].

The MFC/MIC ratio is a critical criterion for the evaluation of the fungicidal activity of antimicrobial agents, and based on the results of the present study, the CuO NPs exhibited significant fungicidal properties with an observed MFC/MIC ratio of 2.6. This finding aligns with those of other research which demonstrated that the high antimicrobial efficacy of CuO NPs attributed to their ability to generate ROS and disrupt microbial cell membranes [ 48 - 51 ]. In contrast to variations observed in MFC/MIC ratios for different antimicrobial agents and NPs, CuO NPs consistently showed a potent fungicidal effect, highlighting their potential to overcome antifungal resistance. This underscores their promise as effective treatments, particularly in scenarios where traditional antifungal therapies may be less effective or compromised by resistance. Future research should explore the broader applicability of CuO NPs, including their long-term effects, optimal dosages, and safety profiles, to fully establish their role in clinical and environmental applications [ 40 ].

Thiruvengadam et al. utilized the aqueous extract of *Millettia pinnata* flowers to synthesize Cu-NPs biologically, demonstrating their promising antioxidant, antibacterial, and anti-inflammatory activities. Characterized by UV-visible spectroscopy, XRD, Fourier-transform infrared spectroscopy (FT-IR), SEM, and transmission electron microscopy (TEM) techniques, the Cu-NPs were confirmed to have a spherical shape with an average size of 23 nm, exhibiting strong efficacy against gram-negative and gram-positive bacteria. Similarly, the current study focuses on the green synthesis of CuO NPs, which displayed significant antifungal activity against M. canis. Both studies underscore the importance of using environmentally friendly, plant-based methods for nanoparticle synthesis, which not only enhance the biological activities of NPs but also offer a sustainable and safer alternative for medical applications. While Thiruvengadam et al. emphasize the multifunctional properties of Cu-NPs, particularly their antioxidant and anti-inflammatory effects, the present study focused on the antifungal potential of CuO NPs, highlighting their applicability in treating dermatophytosis and combating antifungal resistance [ 41 ].

The present study investigated the antifungal efficacy of CuO NPs synthesized via green chemistry against M. canis isolates from dermatophytosis in dogs and cats. The CuO NPs were characterized as having a size range of 10-30 nm with a cubic morphology and high purity. The disk diffusion test showed significant inhibition of fungal growth at concentrations up to 500 ppm. In comparison, the microdilution test revealed MIC values between 500 ppm and 1000 ppm and MFC values between 1000 ppm and 2000 ppm. In contrast, a study performed by Mohamed et al. used *Penicillium chrysogenum* to synthesize zinc oxide and CuO NPs. Their CuO NPs, with a broader size range (10.5-59.7 nm), demonstrated high antimicrobial and antibiofilm activities against various bacteria and fungi. Their study highlighted the broader applications of CuO NPs, including their effectiveness against biofilm-forming bacteria. While both studies confirm the antimicrobial potential of CuO NPs, the present research provided specific insights into their antifungal activity against dermatophytes, which complemented the broader-spectrum findings of the aforementioned study [ 52 ].

Findings of the present research aligned with those of studies carried out by Renuga et al. (2020) and Mohamed et al. (2021) which demonstrated the efficacy of CuO NPs synthesized via a green synthesis protocol. Similar to Renuga et al., who used a plant extract for CuO NP synthesis, the present study employed an eco-friendly approach, highlighting the sustainability and effectiveness of green synthesis methods. Renuga et al. reported high levels of antifungal activity of CuO NPs against *Aspergillus niger* and *Candida albicans*, with an average particle size of 26 nm, confirming the potential of CuO NPs as antifungal agents. The CuO NPs synthesized in the present study exhibited sizes ranging from 10 to 30 nm and showed significant antifungal activity against *M. canis*, particularly at higher concentrations. Results of the present study corroborate the findings of Renuga et al. and Mohamed et al., further validating the effectiveness of green-synthesized CuO NPs in antifungal applications [ 52 , 53 ].

In contrast to the present study, Flores-Rábago et al. investigated CuO NPs produced through an eco-friendly method involving the fungus *Ganoderma sessile*. These NPs were smaller, with average diameters of 4.5 ± 1.9 nm and 5.2 ± 2.1 nm, and were characterized using techniques, such as TEM, FTIR, and XRD. Their study found that CuO NPs demonstrated antimicrobial activity against various bacteria, including *Staphylococcus aureus*, *Escherichia coli*, and *Pseudomonas aeruginosa*. Additionally, they evaluated the biocompatibility of these NPs, finding them to be minimally toxic to mammalian cells at specific concentrations. While both studies emphasized the antimicrobial potential of CuO NPs, the current research focused on fungal infections in veterinary medicine. In contrast, Flores-Rábago et al. explored a broader range of antibacterial applications and assessed biocompatibility [ 54 ]. The differences in NPs size and testing scope highlight the varied potential applications and effectiveness of CuO NPs for treating different types of infections.

A study performed by Habibi et al. and the present research explored the antifungal properties of metal oxide NPs, particularly CuO NPs, against pathogenic fungi. Habibi et al. synthesized magnesium oxide (MgO) and CuO NPs chemically and assessed their antifungal activity against various *Aspergillus* species, finding that CuO NPs exhibited significant inhibitory and fungicidal effects, particularly against *A. niger* and *Aspergillus fumigatus*. The above-mentioned study reported that the NPs had a size range of 10-60 nm and demonstrated a high level of purity, with mean MIC and MFC values of 10.25 and 10.08 mg/mL for CuO NPs, respectively. In contrast, the present study used a green synthesis method to produce CuO NPs, which were found to inhibit *M. canis* effectively. Both studies underscored the potent antifungal activity of CuO NPs. However, Habibi et al. focused on a broader range of *Aspergillus* spp. and chemically synthesized NPs. At the same time, the present study emphasized the eco-friendly green synthesis approach and its application against a specific dermatophytic fungus. Findings of both studies highlighted the potential of CuO NPs as effective antifungal agents, though the synthesis methods and targeted fungal species differ [ 49 ].

A study conducted by Amin et al. and the present study both emphasized the advantages of green synthesis for the production of CuO-NPs and their potential applications as antimicrobial agents. Amin et al. synthesized CuO-NPs using *Aerva javanica* plant leaf extract. They characterized the NPs using XRD, FTIR, and SEM techniques, revealing a crystalline morphology with an average size of 15-23 nm. These NPs demonstrated significant antimicrobial activity against both bacterial and fungal pathogens, with comparable effectiveness to standard medications, like norfloxacin and amphotericin B. Similarly, the present study also utilized a green synthesis approach to produce CuO-NPs, which exhibited notable antifungal activity against *M. canis*. Both studies highlighted the eco-friendly, cost-effective, and scalable nature of green synthesis methods, making them ideal for the generation of NPs with potent antimicrobial properties. While Amin et al. focused on the broad-spectrum antimicrobial efficacy of CuO-NPs, the present study specifically explored their antifungal potential, underscoring the versatility and promise of CuO-NPs in addressing microbial resistance and improving infection treatments [ 48 ].

Muñoz-Escobar and Reyes-López's study on polycaprolactone- CuO NPs (PCL-CuONPs) emphasized the enhancement of antimicrobial properties by incorporating CuO NPs into polymer matrices. The CuO NPs in PCL fibers were characterized as having a diameter of 35 ± 11 nm, with a uniform size distribution observed through dynamic light scattering. The composite PCL-CuONPs demonstrated significant antifungal activity against various *Candida* spp., showing promise for practical antifungal applications. While the present study confirmed the antifungal effect of CuO NPs, it focused explicitly on their effectiveness against dermatophyte infections in the veterinary context. In contrast, a study performed by Muñoz-Escobar et al. explored the broader potential of CuO NPs embedded in polymer matrices, revealing their effectiveness against different fungal species and highlighting the benefits of composite materials. These comparisons underscore the versatility and potential of CuO NPs in various antifungal applications, from veterinary medicine to practical antifungal dressings [ 55 ].

## Conclusion

The present study revealed the promising antifungal potential of CuO NPs against *M. canis*, showcasing their ability to inhibit and kill the fungus. While the antifungal effects observed at the tested concentrations were somewhat lower than those of standard antifungal medications, the possibility of achieving comparable or even superior efficacy with higher doses of CuO NPs remains a compelling avenue for future exploration. Additional research with larger sample sizes and *in vivo* clinical evaluations is essential to solidify these findings. Nevertheless, the consistent outcomes across MIC, MFC, and disk diffusion tests, combined with the urgent need to address drug resistance and the adverse effects of current antifungal treatments, suggest that CuO NPs hold considerable promise as a novel and effective alternative in the fight against fungal infections.
